# Refining scores based on patient reported outcomes – statistical and medical perspectives

**DOI:** 10.1186/s12874-019-0806-9

**Published:** 2019-07-31

**Authors:** Manuel Feißt, André Hennigs, Jörg Heil, Helfried Moosbrugger, Augustin Kelava, Ilona Stolpner, Meinhard Kieser, Geraldine Rauch

**Affiliations:** 1 Institute of Medical Biometry and Informatics, University of Heidelberg, Im Neuenheimer Feld 130.3, D-69120 Heidelberg, Germany; 2 Department of Gynecology and Obstetrics, University of Heidelberg, Im Neuenheimer Feld 440, D-69120 Heidelberg, Germany; 3 Department of Psychology, Johann Wolfgang Goethe University, Theodor-W.-Adorno-Platz 6, D-60323 Frankfurt am Main, Germany; 4 Methods Center, Eberhard Karls University, Hölderlinstr. 29, D-72074 Tübingen, Germany; 5 Charité - Universitätsmedizin Berlin, corporate member of Freie Universität Berlin, Humboldt-Universität zu Berlin, and Berlin Institute of Health, Institute of Biometry and Clinical Epidemiology, Charitéplatz 1, D-10117 Berlin, Germany; 6 Berlin Institute of Health, Anna-Lousia-Karsch 2, D-10178 Berlin, Germany

**Keywords:** Patient reported outcomes, Latent variables, Factor scores, Questionnaire refinement

## Abstract

**Background:**

Patient Reported Outcomes (PRO) are gaining more and more importance in the context of clinical trials. The assessment of PRO is frequently performed by questionnaires where the multiple items of a questionnaire are usually pooled within summarizing scores. These scores are used as variables to measure subjective aspects of treatments and diseases. In clinical research, the calculation of these scores is mostly kept very simple, e.g. by a simple summation of item values. In the medical literature, there is hardly any guidance for performing a refinements of questionnaires and for deducing adequate scores. In contrast, in psychometric literature, there are plenty of more sophisticated methods, which overcome typical assumptions made in traditional (sum) scores, however to the prize of more complicated algorithms, which might be difficult to communicate. When faced with the practical task to refine an existing questionnaire, there exist a clear gap of guidance for applied medical researchers. By this article we try to fill this important gap between psychometric theory and medical application by illustrating our methodological choices on the example of a clinical PRO questionnaire.

**Methods:**

Based on our experiences with the refinement of the BCTOS, a PRO questionnaire to assess aesthetic and function after breast conserving therapy in breast cancer patients, we present the following general steps that we performed by refining the BCTOS questionnaire and its scores: 1. Refinement of the length of the questionnaire and the (item-factor) structure. 2. Selection of the factor score estimation method. 3. Validation of the refined questionnaire and scores with respect to validity, reliability and structure based on a validation cohort.

**Results:**

Our step-step-step procedure helped us to shorten the current form of the BCTOS and to redefine the factor structure. By this, the compliance of patients can be increased and the interpretation of the results becomes more coherent.

**Conclusions:**

We present a step-by-step procedure to refine an existing medical questionnaire along with its scores illustrated and discussed by the refinement of the BCTOS.

**Trial registration:**

Due to the character of the study (no intervention study), no registration was performed.

## Background

In clinical trials, patient reported outcomes (PRO) are gaining more and more importance [1, 2]. PROs can help to measure constructs (e.g., subjective well-being) as latent variables that cannot be examined in an objective way. By this, PROs are particularly appealing if the aim is to assess subjective endpoints which often better reflect the patient’s individual view. The assessment of PRO is frequently performed by questionnaires, where patients respond to various items on an ordinal Likert scale. Thereby, the clinical researcher is often not only interested in assessing one specific outcome, but aims to collect several aspects related to a larger global endpoint. For example, when assessing the global endpoint quality of life, there are several related sub-outcomes such as physical well-being and subjective well-being. As a consequence, related questionnaires tends to be rather long in order to assess as much information as possible (the more aspects are measured, the longer is the questionnaire). However, the demand to answer a large amount of questions increases the patient’s effort and can lead to low response rates and/or bad quality of the answers. Thus, questionnaires should be only as long as necessary to maintain patient’s compliance. Therefore, the selection of adequate items out of a potentially large site of candidate items (item pool) is a very important aspect of questionnaire development.

In general, to achieve a higher reliability and content validity in the measurement of a latent variable, multiple items of a questionnaire are usually combined into a single summarizing score. Especially in clinical research, the calculation of such scores is mostly kept simple. For example, the mean or the sum of the patient’s answers is considered as the final score estimate, sometimes followed by a linear transformation to an easily interpretable scale (e.g. 0–100). However, from a psychometric theory view point, simple summation should be restricted only to scores where the factor loadings resulting from a corresponding factorization are similar or nearly equal, a requirement that is often not fulfilled and hardly ever verified [3]. In the psychometric literature, there are a number of methods that are more complex, but also more appropriate to define such scores.

In conclusion, the selection of adequate items and an appropriate scoring procedure are two main criteria that guarantee a valid and reliable measurement of latent variables which are assessed by PRO. Both aspects are usually ignored in the clinical context, mainly because recommendations and guidance for clinical researchers on how to construct good questionnaires and related scores are still missing.

This lack of guidance was one of the major challenges for us when we were recently confronted with the task to refine the Breast Cancer Treatment Outcome Scale (BCTOS) [4], a PRO questionnaire to assess aesthetic and function after breast conserving therapy in breast cancer patients. Although this questionnaire was used in practical application for years, it can be criticized for being redundant in some aspects. As a consequence, the number of items seems to be too high and the three sub-scores of the BCTOS seem not well separated. When facing the challenge to refine this particular questionnaire, we were confronted with very general aspects of psychometric theory. The task thereby was to find a good compromise between methodologic correctness and feasibility in practical application. As this is a task which goes far beyond the specific goal to refine the BCTOS the aim of this article is to give an insight in our experiences and methodological choices we made during this refinement process. The first steps can already be found in Hennigs et al. [5]. However, the results presented in that paper are more focused on medical aspects and ended with yielding a new item factor structure. The detailed steps in refining and revising a questionnaire with its scores goes far beyond the scope of the former paper. For example, a number of choices and decisions for and against the use of different methodological concepts had to be made. By summarizing our experiences, we derived a step-by-step procedure to refine the BCTOS along with its scores that we want to present in this article. Although the formal methods incorporated in this step-by-step procedure are not new from a psychometric point-of-view, we hope that applied medical researchers and statisticians being faced with a concrete medical questionnaire can learn and benefit from our presented experiences and methodological choices we made by refinement of the BCTOS. By this, the article fills an important gap between psychometric theory and medical application.

## Methods

As an exemplary PRO questionnaire we used the Breast Cancer Treatment Outcome Scale (BCTOS, Stanton et al. [4]) which was designed to assess women’s subjective evaluation of both the aesthetic and functional outcomes after breast conservation surgery for breast cancer patients. These outcomes are directly related to patient’s quality of life [6, 7]. This questionnaire comprises 22 items resulting in three distinct sub-scores assessing the Aesthetic Status, the Functional Status and the Breast Sensitivity Status [8]. Patients are instructed to rate each item of the BCTOS on a four-point Likert scale evaluating the differences between the treated and untreated breast (1 = no difference to 4 = large difference). Therefore, for the resulting factor scores, which are in the original BCTOS version calculated by the mean of the items corresponding to the respective sub-scores, higher score values indicate worse outcome. Practical experiences during years of using the BCTOS made us believe that the BCTOS should be revised [5]. In detail, some of the 22 individual items of the BCTOS seem to be redundant with respect to both wording and discriminatory power, especially for items regarding functional aspects. That might be explained by the substantial evolution of surgical techniques to less invasive procedures in breast conserving surgery [9]. Furthermore, the interpretation of the third subscale, i.e. the Breast Sensitive Status, in the context of aesthetic and functional outcomes is not straightforward and rises difficulties. Therefore, our aim was to create a tool that creates only two scores, one concerning the aesthetic and one concerning for the functional outcomes after breast conserving surgery. However, in an refined version of the BCTOS, the information assessed by the previous Breast Sensitive Status should not be deleted but only be rearranged in a more intuitive way.

With respect to these points, we performed a refinement of the original version of the BCTOS. The process of refining the BCTOS was performed together with physicians from the Department of Gynecology and Obstetrics of the University of Heidelberg. It was based on an a retrospectively recruited test data set (collected between 2007 and 2012), consisting of the data of 871 patients who underwent breast conservation therapy [5]. In addition, we prospectively collected a validation data set comprising the refined version of the BCTOS consisting of 203 patients recruited between June 2017 and May 2018.

All analyses are performed using the statistic software R Version 3.5 or higher [10], using the packages “psych” [11, 12], “lavaan” [12] and “MBESS” [13].

We present a three-step procedure to refine the BCTOS questionnaire based on the above mentioned existing test data set and a validation data set. This procedure was developed in context of the BCTOS, however we hope that our experiences and methodological choices will help applied medical researchers and statisticians by the refinement of other PRO questionnaires which are in need of improvement. In general, the test data set used for refinement of the questionnaire must be representative for the patient population of interest and should be adequately large depending on the length of the questionnaire and the number of (sub-) scores the researcher is interested in. The validation data set should ideally be prospectively collected. Alternatively, the test data set can be split into a test and a validation set. However, the observed results from the original potentially long questionnaire may deviate from the results of a shorter refined version and therefore we encourage to use a prospective validation cohort. We divided our refinement procedure for the BCTOS questionnaire into the following general steps:

1. Refinement of the length of the questionnaire and the (item-factor) structure based on a test data set
2. Selection of the factor score estimation method
3. Validation of the refined questionnaire and score with respect to validity, reliability and structure on a validation cohort.

## Results

### Step 1: refinement of the length and structure

Before starting the refinement procedure, it is very important that the physician really has a deep understanding of what she or he intends to measure. Then, as a first step in our proposed refinement procedure, the structure and the related lengths of the questionnaire should be analyzed and then refined. The optimal length of the questionnaire depends on among other criteria on the choice of the factor structure. Therefore, these two aspects should be addressed in conjunction. First, we applied a factorization algorithm based on the new selected items to analyze the underlying structure by applying an exploratory factor analysis. Thereby, we used the polychoric correlation coefficient for the correlation between the different items, since this corresponds to the most exact method to estimate correlations for ordinal variables [14]. In the software R, this can be performed by the “fa” function from the “psych” package. To do so, the estimation method (e.g. maximum likelihood, minimum residuals) and the rotation method (e.g. orthogonal, oblique) must be chosen. There exist a high number of factor methods where each of the methods has its advantages and disadvantages [15]. In our case, we decided to use the minimum residual method since it gives robust estimates even for poor, skewly distributed items, which is a common feature of questionnaires items [15]. Afterwards, a factor rotation method has to be determined in order to get interpretable factor loadings. Since we assumed our resulting factors to be correlated, because they refer to related clinical aspects, we opted for an oblique rotation method for the BCTOS.

In the next step, we applied several methods to determine the number of underlying factors (i.e. Scree-Plot, Kaiser-criterion, parallel-analysis [16]) and compared them in order to guarantee a robust choice of the number of factors. In our case, the different criteria recommended different numbers of factors for the BCTOS-questionnaire: The Kaiser Criterion and the scree plot analysis suggested a two factor solution whereas the parallel analysis suggested a four factor solution. Since, from a clinical point of view, it was our aim to get a questionnaire with only two scores (one for aesthetic and one for function) we opted for the two factor solution. With respect to item selection, items that do not load distinctly on one of the single factors (distinct factor loading structure per item: one factor loading > 0.4 and the other factor loadings < 0.3 [8]) were first candidates that possibly can be dropped from the pool of candidate items. In our application, the items “breast pain”, “ability to lift objects” and “fit of shirt sleeve” were excluded from the item pool due to not showing distinct factor loadings.

To further reduce the subset of items, there we found two important perspectives. First, the physician experienced in the field must judge the importance and the potential redundancy of all items from a clinical perspective. Second, from a statistical point of view we identified possibly redundant items by assessing item difficulty, item variance and item-total correlation. Item difficulty is the mean of all patient’s answers on this specific item and item variance is the respective variance. These two parameters are strongly related. Items with very low or too high item difficulty have small item variance and are candidates to be dropped. The item-total correlation measures the correlation between the item answer of a patient and its respective sum score (without the specific item). Items with low or negative item-total correlation are further candidates that possibly can be dropped. Furthermore, we investigated pair-wise polychoric item correlations, where we dropped one of two highly correlated items or pooled both items into one subsuming item. In detail, the item “fit of clothing” was dropped due to redundancy regarding the item “fit of bra”. Furthermore, due to high correlations, the items “shoulder movement”, “shoulder stiffness” and “shoulder pain” were pooled into the new item “shoulder discomfort”. Moreover, the items “arm movement”, “arm pain” and “arm stiffness” were condensed into the new item “arm discomfort”.

When choosing the most relevant subset of items, we made sure that each (sub-)score contained a sufficient number of items depending on the homogeneity of the latent variable to ensure an adequate reliability [16]: for illustration, for the homogeneous latent variable “function” of the BCTOS a small number of 4 items appears to be sufficient, whereas for the multifaceted latent variable “aesthetic” a larger number of items (i.e. 8) is necessary to ensure an adequate measurement.

**Fig. 1:**
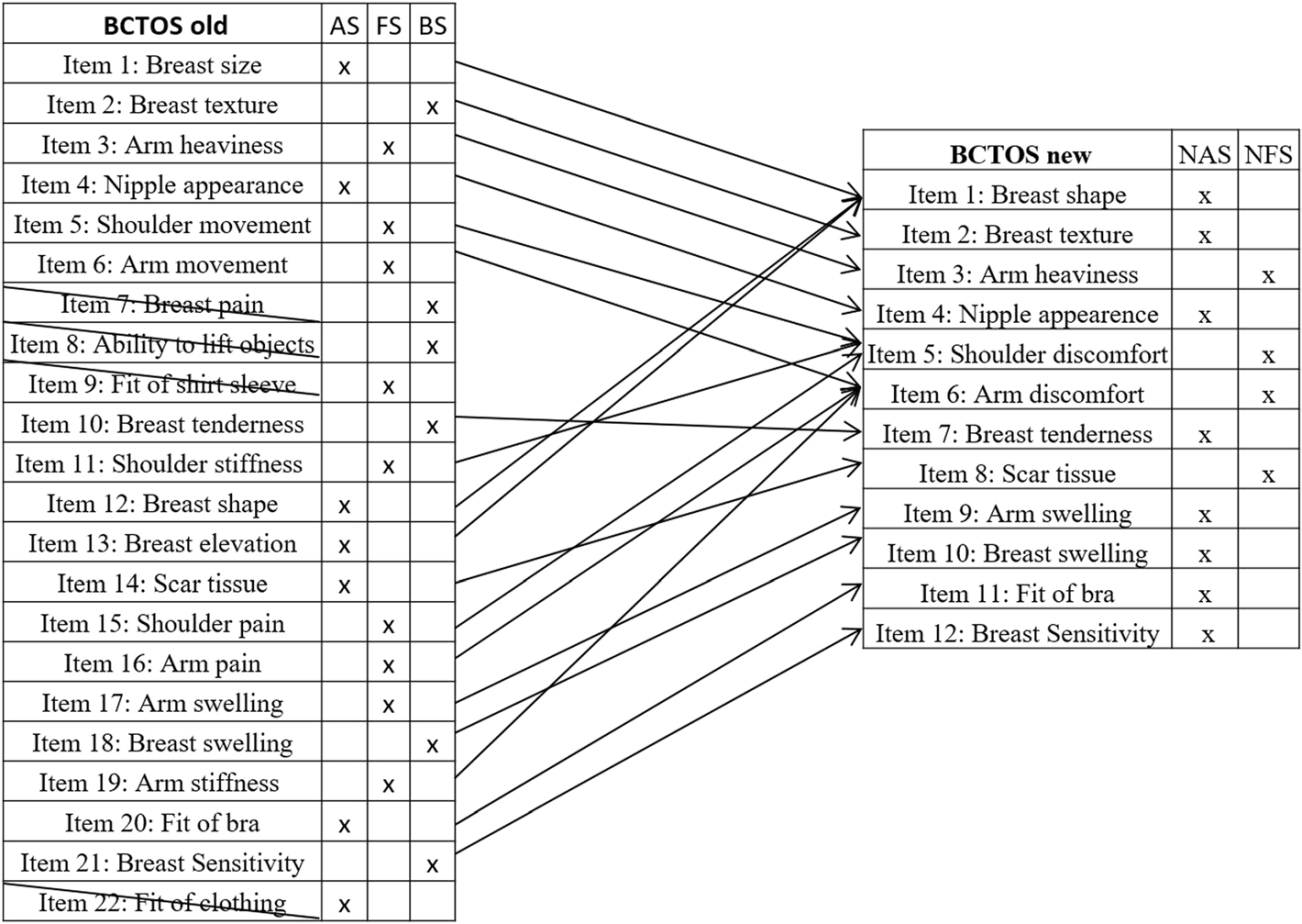
Condensation of old BCTOS into BCTOS-12 (BCTOS new) legend: Condensation of old BCTOS into BCTOS-12 (BCTOS new) with former and new item assignments to the scores Aesthetic Status (AS), Functional Status (FS), Breast Symptoms (BS), New Aesthetic Status (NAS) and New Functional Status (NFS)

In conclusion, we obtained a shorter version of the BCTOS with 12 items on two scales, referred to as the BCTOS-12. The condensation of the former BCTOS into the BCTOS-12 can be found in Fig. 1 and is discussed in further detail in Hennigs et al. 2018 [5].

After these steps, we repeated the exploratory factor analysis on 10000 bootstrapped samples from the reduced item set (sampling with replacement). This step was implemented in R and can be realized via the package “boot”. This step is required to evaluate the robustness of the new item factor. In our application, we mimicked the answers of new combined items within the existing test data set, by calculating the corresponding integer-rounded mean of the original item values. As a kind of “sensitivity” analysis we repeated this procedure with the minimum, maximum and the median as the mimicked item values and found no notable differences to the integer-rounded mean approach. Although in the literature a relative small number of bootstrap samples appears to be sufficient, since there is no big calculation effort in increasing the number of samples, we decided to generate always more than 1000 bootstrap samples [17]. We determined beforehand that if a notable (>%5, [16]) proportion of the bootstrapped samples do not confirm the factor structure (i.e. showing the same item factor assignment as proposed with distinct factor loadings; criterion for distinct factor loadings per item: one factor loading > 0.4 and the other factor loadings < 0.3, see above) the current item factor combination should be carefully reflected and the identified meaningful subset of items should potentially be redefined again together with the physician. Therefore, we would overthink the elimination of items and we would try to find a more robust item factor structure, e.g. by re-introduce eliminated items. However, concerning the BCTOS-12, 99.5% of the bootstrap samples confirmed the distinct item factor structure indicating a high robustness of the item factor structure.

### Step 2: selection of the factor score estimation method

In the next step, we determined a score computation method corresponding to the identified factors. In our application, we need to find scores for the Functional Status and the Aesthetic Status.

Factor score computation methods can be distinguished into two general approaches - refined and non-refined methods.

In clinical applications, most often non-refined methods are used, which are in the most cases simple (unweighted) summation (or means) of the item outcomes. Unweighted summation implies that every item is equally influenced by the latent construct, which is wrong when the factor loading of items differs notably. The score computation of the original BCTOS is based on unweighted summation, however, in the shorter version of the BCTOS, our proposed BCTOS-12 showed different factor loadings for the single items in the test data set as well as for the validation data set.

As an alternative to using simple summation, weighted sum scores are also proposed in the literature [18] and are still considered as non-refined scoring methods. The weights can thereby be determined, e.g. by clinical experts or by a patient’s judgment.

In contrast to the non-refined methods, the refined factor scores (e.g. Regression Scores, Bartlett Scores, Anderson Rubin Scores) are based on linear combinations of the observed variables. They are simultaneously calculated for all patients of the data set and, therefore, the resulting patients’ score is depending on the underlying dataset. Thereby, the aim is to “[ …] consider what is shared between the item and the factor and what is measured” [19]. Refined factor scores can be computed in R with the function “fa” from the “psych” package. Generally, refined methods are computationally more complex than the non-refined methods but also more valid, i.e. they give more accurate estimators of each patient’s “true” level of the underlying latent variables. Based on these considerations, a patient’s score can differ if the item responses of the other patients are changing. This problem is the same for the (weighted) summation approaches, with weights given as factor loadings as the value of the respective factor loading is also depending on the underlying dataset.

In our application, we calculated Pearson correlation coefficients between the different factor score computation methods and the original unweighted mean factor scores for the BCTOS-12. Despite the fact that the factor loadings of the BCTOS-12 were considerably different, we found extremely high correlations (> 0.95) between all resulting factor scores estimators based on the different approaches. From a methodologic point of view, the refined scoring methods are superior to sum or mean scoring methods. However, because of the detected extremely high correlations we opt for the much easier mean score as the factor score estimation method of choice. Since the mean score is a lot of easier to handle, we thereby hope to further encouraging the establishment of the BCTOS in clinical research.

In summary, the advantages and challenges of using refined or non-refined methods should be carefully compared for the application at hand. Based on our considerations, we saw many practical advantages by maintaining the initial scoring method of the original questionnaire.

### Step 3: validation of the refined questionnaire and scores

After the determination of the item factor structure and the score computation method, we tested the validity, reliability and structure of the BCTOS-12 by a prospectively collected validation cohort. Since PRO tend to show high variability, we recruited a relatively large validation cohort with a sample size > 200 [20] in order to achieve reasonable estimators for reliability and validity.

There are different ways to examine the validity of a questionnaire (e.g. content/divergent validity, construct validity, criterion validity). For clinical PRO questionnaires, a widely used approach is to calculate the construct validity by examining the convergent and divergent validity, where the scores of the refined questionnaire are compared to a validated reference questionnaire measuring related aspects. This is done by assessing correlations between the new factor scores and the scores of the reference questionnaire. A questionnaire provides a high validity if its factor scores are reasonably correlated to the reference scores of the (validated) reference questionnaire.

To perform the validation step, we additionally collected data of the EORTC QLQ C30 BR23 (European Organization for Research and Treatment [21]), a cancer-specific quality of life (QoL) questionnaire, as a reference questionnaire. The EORTC QLQ C30-BR23 consists of a base module of 30 items (C30) and a breast cancer-specific addendum of 23 items (BR23), based on ordinal rating scales [21, 22]. The items are summarized in several sub-scores representing different aspects of QoL. Spearman’s rank correlation coefficients between the new scores of the BCTOS-12 and the scores of the QLQ C30 BR23 were calculated. The results can be seen in Table 1. The scores showed a reasonable convergent and divergent validity, e.g. the new aesthetic score showed high correlations to the “Body image” and the “Breast symptoms” score (− 0.45, 0.71), the new functional score showed high correlations to the “Arm symptoms” and the “Physical functioning” score (0.77, − 0.55) and, in contrary concerning the divergent validity, both scores showed very small correlations to the “Fatigue” and the “Diarrhea” score (0.05, − 0.01, 0.06, 0.03).

**Table 1:** Spearman’s rank correlation coefficients

EORTC scale	Aesthetic Scale	Functional Scale
Physical functioning	−0.48	− 0.55
Role functioning	−0.54	− 0.47
Emotional functioning	−0.46	− 0.33
Cognitive functioning	−0.31	− 0.36
Social functioning	−0.47	− 0.45
Fatigue	0.05	−0.01
Nausea and vomiting	0.2	0.2
Pain	0.53	0.55
Dyspnoea	0.19	0.31
Insomnia	0.42	0.2
Appetite loss	0.35	0.32
Constipation	0.23	0.19
Diarrhoea	0.06	0.03
Financial difficulties	0.25	0.31
Global health status	−0.56	−0.48
Systematic therapy side effects	0.38	0.41
Upset by hair loss	0.1	0.11
Breast symptoms	0.71	0.48
Arm symptoms	0.41	0.77
Body Image	−0.45	−0.31
Sexual functioning	−0.11	− 0.15
Sexual enjoyment	−0.12	− 0.22
Future perspective	−0.29	− 0.27

Legend: Spearman’s rank correlation coefficients between the new scores of the BCTOS-12 (columns) and the scores of the QLQ C30 BR23

A fast and widely used way to get a measure for the reliability of the new questionnaire is to assess the internal consistency. We preferred the use of McDonald’s Omega rather than the widely used Cronbach’s alpha, since Cronbach’s alpha is based on the assumption of equal factor loadings and furthermore, McDonald’s Omega can be used multidimensional, too [23]. As for Cronbach’s alpha, McDonald’s Omega values > 0.8 can be interpreted as a good internal reliability. For the BCTOS-12, we obtained McDonald’s Omega of 0.888 and 0.900 indicating a good internal consistency for the two scores of the BCTOS-12. Since we assumed correlated factors, we also considered the multidimensional McDonald’s Omega coefficient which confirmed the good reliability of our questionnaire (Omega total = 0.908, Omega hierarchical = 0.902). Thereby, we calculated the McDonald’s Omegas based on polychoric correlations, which again can be easily computed in R in the “omega” function of the package “psych”.

To test the new item-factor structure, we performed a confirmatory factor analysis [24] and analyzed its model fit. For the estimation of the parameters of the confirmatory factor analysis, again an estimation method has to be determined (e.g. robust maximum likelihood, weighted least squares, …). Since most PRO questionnaires in clinical research have ordinal scaled item variables, we used the weighted least squares method, since it is distinguished as one of the most appropriate approaches for structural equation modeling with ordinal observed variables, because this methods assume that continuous latent variables were “coarsely categorized by the measurement process to yield the observed ordinal variables, and that the model proposed by the researcher pertains to these latent variables rather than to their ordinal manifestations” [25]. Furthermore, we found that robust maximum likelihood and diagonally weighted least square result in similarly appropriate results [26].

There exists a number of different model fit measures by which the goodness of fit can be evaluated. In general, if we compare a new model with an existing model, we can compare the model fits by a single fit parameter to identify the better model. However, if there is no comparable model, the model fit should be examined by different descriptive measures which can be compared to different cutoffs from the literature. In the literature it is recommended to use several indices simultaneously to represent different classes of goodness of fit criteria [27]. If not all of the presented fit measures meet their respective cutoff, the strength of violation and the possible impact on the model selection has to be discussed. In order to prevent a strong violation of the cutoffs as late as in Step 3, we already calculated the fit indices in Step 1 based on the test data set and took into account the results in the refinement procedure of the item-factor-structure in Step 1 (see above and in Table 2). We opt for the following fit indices because they are widely used and recommended from the literature [16, 28–30]:

1. A root mean square error of approximation (RMSEA) < 0.05 (acceptable fit ≤0.08) [16, 27]: The RMSEA is an index of the difference between the observed covariance matrix and the hypothesized covariance matrix of model.
2. A comparative fit index (CFI) > 0.97 (≥0.95 acceptable) [16, 27]: Similar to the RMSEA the CFI examines the discrepancy between the data and the hypothesized model and additionally adjusts for the sample size.
3. A Tucker-Lewis index (TLI) > 0.95 (≥0.90 acceptable) [16, 27]. The TLI analyzes the discrepancy between the hypothesized and null model (simplest model) referring to the chi-squared value.

**Table 2:** Model fit measures of the confirmatory factor analysis and its multidimensional extensions

Test data	RMSEA	CFI	TLI	Validation data	RMSEA	CFI	TLI
Standard	0.069	0.963	0.954	Standard	0.103	0.974	0.968
Hierarchic	0.07	0.963	0.953	Hierarchic	0.105	0.974	0.967
Bifaktor	0.047	0.987	0.979	Bifaktor	0.083	0.987	0.979

Legend: Modell fit measures (*RMSEA* root mean square error of approximation, *CFI* comparative fit index, *TLI* Tucker-Lewis index) of the confirmatory factor analysis (Standard) and its multidimensional extension (Hierarchic, Bifaktor) of the test data (existing) and the validation data (prospective collected)

If the underlying factors of the refined questionnaire show high correlations, higher order factor models (e.g. bifactor, general factor models [31–33]) can improve the model fit and strengthen the use of the questionnaire as one single tool (instead of several different questionnaires, with separate scoring procedures).

Concerning the BCTOS-12, we used a standard confirmatory factor analysis as well as higher-order models (hierarchic and bifactor model) to account for the correlation between the factors. These were based on the test dataset from step 1 and step 2 and based on the validation cohort dataset, as well. The best model fit was found for the bifactor model (see Table 2). The preference of the bifactor model strengthens the use of the questionnaire as a single tool and thus, indicating the calculation of its scores to be based on all items of the questionnaire. However, as shown above, the refined scores in the example of the BCTOS-12 are highly correlated to the mean scores and in addition, the mean scores are a lot easier to handle. Therefore, we finally decided to maintain the initial scoring method of mean calculation. However, these findings may provide the basis for the development of a summary score comprising all items of the questionnaire. Since the model fit measures indicate an acceptable model fit for all of the tested models, we considered the new two dimensional structure of the BCTOS-12 to be verified.

## Discussion

In this paper we presented a three-step procedure for the refinement of the Breast Cancer Treatment Outcome Scale (BCTOS [4]). The BCTOS is a PRO questionnaire which was designed to assess women’s subjective evaluation of both the aesthetic and functional outcomes after breast conservation surgery for breast cancer patients.

Our presented procedure of the original BCTOS resulted in a shorter and more straightforward version, the BCTOS-12. Based on only 12 items, the new version it is shorter and therefore more comfortable to handle for physicians and patients. The refined questionnaire comprises exactly two subscales: one subscale regarding to the breast area concerning more aesthetic aspects and one subscale concerning the arm and shoulder are regarding to more functional aspects. Thus, the interpretation of the subscales is more straightforward than the interpretation of three subscales in the original version.

The aim of this article is to give an insight in the methodological choices we made in order to give medical researchers and statisticians being faced with the same problem of the refinement of a PRO questionnaire some orientation and guidance. However, the choice of the underlying methodology should always be considered individually for the questionnaire and the medical research field at hand. To illustrate the general points and problems to which one is confronted during the refinement process of a questionnaire, we tried to formalize the methodologic steps required. However, to give a full and complete guidance for all possible methodologic aspects of a questionnaire is a very complex task and would go beyond the scope of this article. Nonetheless, we did our best to provide the interested reader with various references where a lot of further information could be found.

Refinement of PRO questionnaires and scores is both – a statistical and a medical task. We made the experience that the restriction to statistical aspects of the procedure alone is not sufficient for a comprehensive refinement of a questionnaire. Therefore, we recommend the cooperation between physicians and statistician, to join their clinical and methodologic knowledge and experience.

However, the statistical has a strong impact. We tried to give an insight in the existing methods and recommendations for the choice and determination of the required statistical key methods and parameters. We thereby took into account the actual state of the art in the field of psychometrics. For example, we used McDonald’s Omega instead of Cronbach’s alpha as a measure of the internal consistency. Similarly, we found polychoric correlations to be a more complex but better approach for the calculation of correlations between ordinal variables than Spearman’s rank correlation coefficient [34].

Furthermore, we analyzed various factor score estimation methods. From a statistical point of view, the refined methods are superior to non-refined methods, e.g. refined methods give more accurate estimators for the factor scores with less bias than scores based on (weighted) sum scores. However, they are not easy to compute, compared to a simple summation algorithm, and they have to be based on a notably large data set to guarantee reliable estimators. Therefore, these methods are currently only rarely applied in a clinical research. However, in our application, we found extremely high correlations between the refined factor score estimators and the mean factor score estimators, indicating the additional benefit for using these complex methods can be moderate in specific applications. Therefore, to encourage the use of the BCTOS-12 in clinical practice, we prefer to rely on the mean factor scores estimators for the BCTOS-12 subscales.

In general, one should bear in mind that the procedure of refining a questionnaire is a task that is financially expensive and time consuming. Especially the necessary recruitment of a validation cohort, resulted in a great financial effort and a high time requirement. Nonetheless, this effort is necessary, since there is an increasing use of PROs in medical research and it gets more and more important to have reliable questionnaires with appropriate scores for measuring subjective latent factors.

## Conclusion

In this work, we illustrated a possible step-by-step procedure to refine an existing questionnaire with its scores by a clinical PRO example. Psychometrics offers a huge amount of tools for the adequate refinement of questionnaires that are waiting to be used in the field of medical research.

We hope this paper can contribute to bring the methodology of clinical research with PRO on the “next level”.

## Data Availability

The datasets used and/or analyzed during the current study are available from the corresponding author on reasonable request.

## Abbreviations

BCTOS: Breast Cancer Treatment Outcome Scale
CFI: Comparative fit index
EORTC: European Organization for Research
PRO: Patient reported outcomes
QLQ: Quality of life questionnaire
QoL: Quality of life
RMSEA: Root mean square error of approximation
TLI: Tucker-Lewis index
